# Increased cytokine/chemokines in serum from asthmatic and non-asthmatic patients with viral respiratory infection

**DOI:** 10.1111/irv.12155

**Published:** 2013-08-21

**Authors:** María J Giuffrida, Nereida Valero, Jesús Mosquera, Melchor Alvarez de Mon, Betulio Chacín, Luz Marina Espina, Jennifer Gotera, John Bermudez, Alibeth Mavarez

**Affiliations:** a Facultad de Medicina, Instituto de Investigaciones Clínicas “Dr. Américo Negrette”, Universidad del ZuliaMaracaibo, Venezuela; b Servicio de Enfermedades del Sistema Inmune y Oncología, Hospital Universitario “Príncipe de Asturias”, Universidad de AlcaláMadrid, España

**Keywords:** Asthma, chemokines, interleukins, viral lung infection

## Abstract

**Background:**

Respiratory viral infections can induce different cytokine/chemokine profiles in lung tissues and have a significant influence on patients with asthma. There is little information about the systemic cytokine status in viral respiratory-infected asthmatic patients compared with non-asthmatic patients.

**Objectives:**

The aim of this study was to determine changes in circulating cytokines (IL-1β, TNF-α, IL-4, IL-5) and chemokines (MCP1: monocyte chemoattractant protein-1 and RANTES: regulated on activation normal T cell expressed and secreted) in patients with an asthmatic versus a non-asthmatic background with respiratory syncytial virus, parainfluenza virus or adenovirus respiratory infection. In addition, human monocyte cultures were incubated with respiratory viruses to determine the cytokine/chemokine profiles.

**Patients/Methods:**

Patients with asthmatic (*n* = 34) and non-asthmatic (*n* = 18) history and respiratory infections with respiratory syncytial virus, parainfluenza, and adenovirus were studied. Healthy individuals with similar age and sex (*n* = 10) were used as controls. Cytokine/chemokine content in blood and culture supernatants was determined by ELISA. Monocytes were isolated by Hystopaque gradient and cocultured with each of the above-mentioned viruses.

**Results:**

Similar increased cytokine concentrations were observed in asthmatic and non-asthmatic patients. However, higher concentrations of chemokines were observed in asthmatic patients. Virus-infected monocyte cultures showed similar cytokine/chemokine profiles to those observed in the patients.

**Conclusions:**

Circulating cytokine profiles induced by acute viral lung infection were not related to asthmatic status, except for chemokines that were already increased in the asthmatic status. Monocytes could play an important role in the increased circulating concentration of cytokines found during respiratory viral infections.

## Introduction

Viral respiratory tract infections can have profound effects on asthma.^1^ Respiratory viral infections can have a significant influence on asthmatic patients, where viral respiratory infections are found in association with asthma exacerbations.^1^,^2^ The infections associated with these wheezing events are multiple and include infections by respiratory syncytial virus (RSV), human rhinovirus, metapneumovirus, parainfluenza, coronavirus, and other viruses. Previous studies have shown differences in the bronchoalveolar lavage cytokine profiles between non-asthmatic and asthmatic patients related to the type of cellular infiltrate observed,^3^ suggesting that immune response is related to atopic status. However, circulating cytokine response after viral respiratory infection in asthmatic and non-asthmatic status has been little studied. In this regard, circulating blood levels of several cytokines have been reported to be increased during viral respiratory tract infections.^2^ These cytokines may represent inflammatory markers with different profiles in asthmatic and non-asthmatic patients. Several cytokines have been described to play an important role in the pathogenesis of asthma: IL-4, IL-5, IL-8, IL-10, IL-12 (p40), IL-13, IL-17, TNFα, IL-1, MCP-1, RANTES, GM-CSF, eotaxin, and IFNγ. Asthma is one of the most heterogeneous respiratory diseases and may also demonstrate systemic patterns beyond the respiratory system. Determination of serum cytokine levels in asthmatic patients could have potential utility in the diagnosis of asthma and certain phenotypes, in prediction of attacks and choice of treatment. In addition to cytokine/chemokine profiles, the increased systemic concentration of those molecules could activate circulating leukocytes with further deleterious effects in the lung.^1^,^2^ Therefore, the aim of this study was to describe changes in several inflammatory cytokines (IL-1β, TNF-α), Th2 cytokines (IL-4, IL-5), and chemokines as MCP-1 (monocyte chemoattractant protein-1) and RANTES (regulated on the activation normal T cell expressed and secreted) in the systemic circulation during acute viral infection in patients with an asthmatic and a non-asthmatic background and their relationship with the respiratory infection type (upper and lower) and type of virus infection. In addition, to determine the role of monocytes in circulating cytokine profiles, the cytokine/chemokine profiles after viral-monocyte interaction were studied.

## Materials and methods

### Patients

Male and female patients (*n* = 52) presenting clinical diagnosis of acute respiratory infection (ARI; 34 asthmatic patients and 18 without pre-existing asthma) were studied. The inclusion criteria were those individuals who had at least one respiratory symptom, such as cough, wheeze, running nose, or sneeze, and who were suspected by a professional physician to have viral infection. Asthmatic patients were selected according to the criteria of the Global Initiative for Asthma (GINA) Program.^4^ Asthmatic crisis was not present (at least 1 month previous) when blood samples were taken. In addition to suggestive clinical diagnosis (pneumonia or bronchitis), viral infection (RSV, parainfluenza and adenovirus) was confirmed by the presence of virus in specimens from nasopharynx and bronchoalveolar lavage. Viral replication was demonstrated in HEp-2 cell cultures (protocol -520-I; National Institute for Health, USA).^5^ Healthy individuals with similar age and sex (*n* = 10) were studied as controls. Blood samples were obtained from patients and controls, and serum was stored at −70°C until use. We excluded individuals who had cardiac disease, immunodeficiency, and chronic inflammatory disease. No patients were treated with antibiotics, anti-alergics, or steroid when blood samples were obtained. The Ethics Committee of Instituto de Investigaciones Clínicas Dr Américo Negrette and FONACIT (Caracas, Venezuela) approved this study, and written informed consent was obtained from all patients and controls prior to blood collection.

### Respiratory virus preparation

Nasopharynx and bronchoalveolar samples from patients were sonicated and centrifuged, and supernatants were added to HEp-2 cell cultures. Previously, cells were grown to 50% confluence in Eagle's minimum essential medium (MEM) containing 7% FBS and 1% antibiotic/antimycotic. After two washes with PBS, 200 μl of supernatant was added to cell cultures. Cultures were incubated for 1 hour, and then, 300 μl of MEM containing 10% FBS and 1% antibiotic/antimycotic was added. Cultures were then incubated at 37°C in 5% CO_2_ for 96 to 120 hours. Supernatants from cultures were centrifuged and stored at −70°C as the source of virus. Viral cell culture infection (RSV, parainfluenza 1, 2, and 3 and adenovirus) was determined by direct immunofluorescence using a commercial kit (Light Diagnostics SimulFluor Respiratory Screen Kit; Chemicon Internacional, Temecula, CA, USA).

Quantification of serum IgE and IL-1 β, TNF-α, IL-4, IL-5, IL-8, MCP-1, and RANTES in serum and culture supernatants IL-1 β, TNF-α, IL-4, IL-5, MCP-1, and RANTES contents was measured using a commercially available ELISA kits (TNF-α, IL-4 and IL-5; Diaclone, Fleming, France; MCP-1; Endogen, IL, Rockford, USA; IL-1 β and RANTES; Alpco Diagnostics, Salem, NH, USA; IL-8; R & D System, Minneapolis, MN, USA), and the results were expressed as pg/ml in samples from serum and pg/mg of protein from culture supernatants. Serum IgE content was determined by ELISA (Calbiochem Inc., San Diego, CA, USA).

### Monocyte cultures

Mononuclear leukocytes were obtained from heparinized venous blood from five healthy adult donors. Cells were isolated through density gradient centrifugation in Hystopaque 1·077 (Sigma Chemical Co., St. Louis MO, USA). Cells were suspended at 2 × 10^6^ cell/mL in RPMI 1640 supplemented with 100 U/ml penicillin, 10 μg/ml streptomycin, and 10% FBS and then incubated at 37°C in a humidified atmosphere with 5% CO_2_. After 3-hour incubation, adherent cells were enriched by washing away unattached cells twice. After washing, a mean of 3 × 10^5^ adherent cells/well was obtained. Monocyte cultures from each donor were infected (MOI: 1) by virus obtained from each infected HEp-2 cell culture supernatants (RSV, parainfluenza and adenovirus).^6^ Monocytes cultured in HEp-2 cell culture supernatants without virus were used as negative controls. After 24 hours, supernatants were collected and cytokine and chemokine determinations were performed using ELISA. Results were expressed as pg/mg of cellular protein. Monocyte virus infection was determined by direct immunofluorescence (Light Diagnostics SimulFluor Respiratory Screen Kit; Chemicon Internacional, CA, USA).

### Statistical analysis

Values were expressed as mean ± standard deviation. The significance of differences was tested by anova and the Bonferroni *post hoc* test. Correlation analysis was performed using Pearson's correlation. Two-tailed *P* values <0·05 were considered statistically significant.

## Results

**Figure 1:**
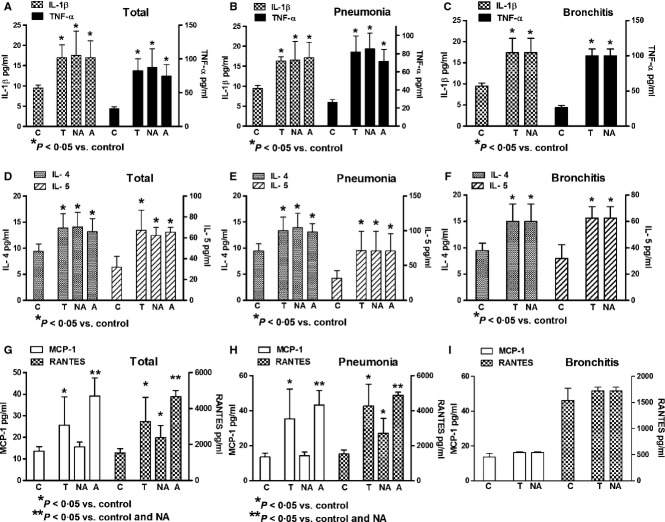
Interleukin-1 beta (IL-1β), tumor necrosis factor alpha (TNF-α), interleukin-4 (IL-4), interleukin-5 (IL-5), monocyte chemoattractant protein-1 (MCP-1) and regulated on activation normal T cell expressed and secreted (RANTES) expressions in non-asthmatic (NA) and asthmatic (A) patients with viral acute respiratory insufficiency. Total values of cytokines (A, D, and G). Patients affected by viral pneumonia (B, E, and H). Patients affected by viral bronchitis (C, F, and I). T**,** Total values; C, Healthy controls. All information in the figures represents the same patients classified according to different statuses.

**Figure 2:**
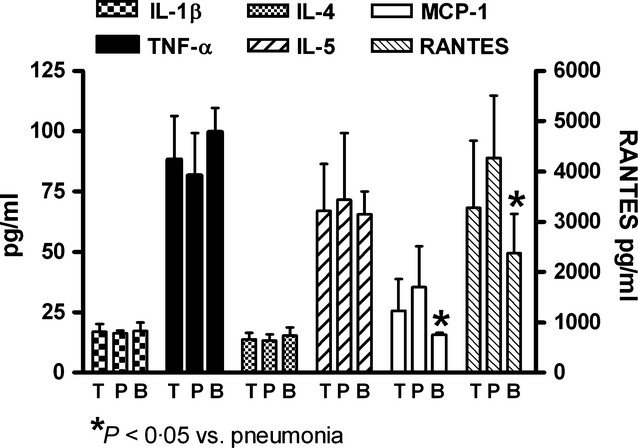
Interleukin-1 beta (IL-1β), tumor necrosis factor alpha (TNF-α), interleukin-4 (IL-4), interleukin- 5, monocyte chemoattractant protein-1 (MCP-1) and regulated on activation normal T cell expressed and secreted (RANTES) expressions in total patients (T), patients with viral bronchitis (B), and viral pneumonia (P). All information in the figures represents the same patients classified according to different statuses.

**Figure 3:**
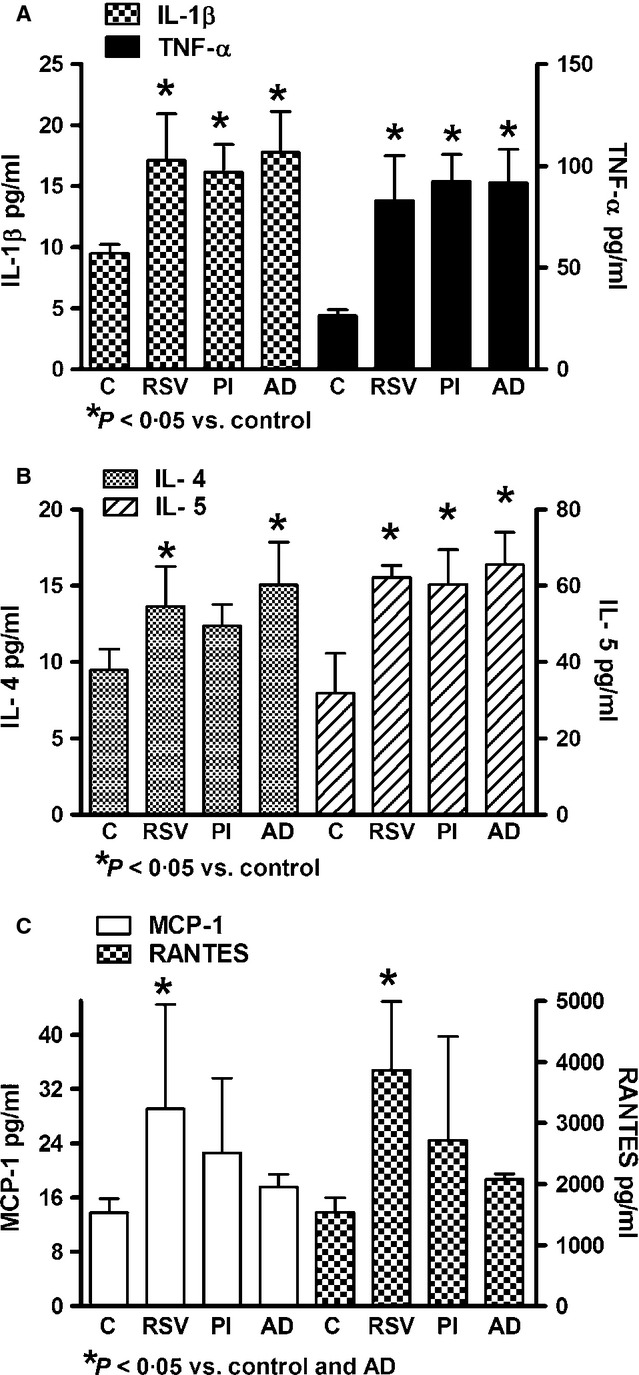
Citokine/chemokine distribution according to type of viral infection. C: Healthy control; RSV: respiratory syncytial virus; PI: parainfluenza virus; AD: adenovirus.

Age and laboratory findings in studied groups are shown in Table 1. Increased levels of IgE were observed in asthmatic patients when compared with non-asthmatic patients and controls. In general, the serum levels of IL-1β, TNF-α, IL-4, IL-5, MCP-1, and RANTES were increased in patients with acute viral respiratory infections (Figure 1). Cytokine expression was similar in asthmatic and non-asthmatic patients; only chemokines (MCP-1 and RANTES) were significantly increased in asthmatic patients when compared with control and non-asthmatic patients. Asthmatic patients with bronchitis were not found (Figure 1). In general, serum chemokines (MCP-1 and RANTES) had lower levels in bronchitis than in pneumonia (Figure 2). The infection by RSV, parainfluenza virus, and adenovirus had similar cytokine induction; however, only RSV induced significant increased expression of chemokines (Figure 3).

**Table 1:** Laboratory findings in non-asthmatic, asthmatic, and control groups

Parameters	Non-asthma	Asthma	Controls
Age (years)	12–77	13–70	15–72
Serum total IgE (IU/ml)	56·80 ± 9·29	380·50 ± 13·22*	18·49 ± 5·51
Percentage of blood eosinophils	2·66 ± 1·21	6·16 ± 4·07	2·0 ± 0·70

* *P* < 0·01 vs control and non-asthmatic patients. Non-asthma (*N* = 34); Asthma (*N* = 18); Control (*N* = 10).

**Figure 4:**
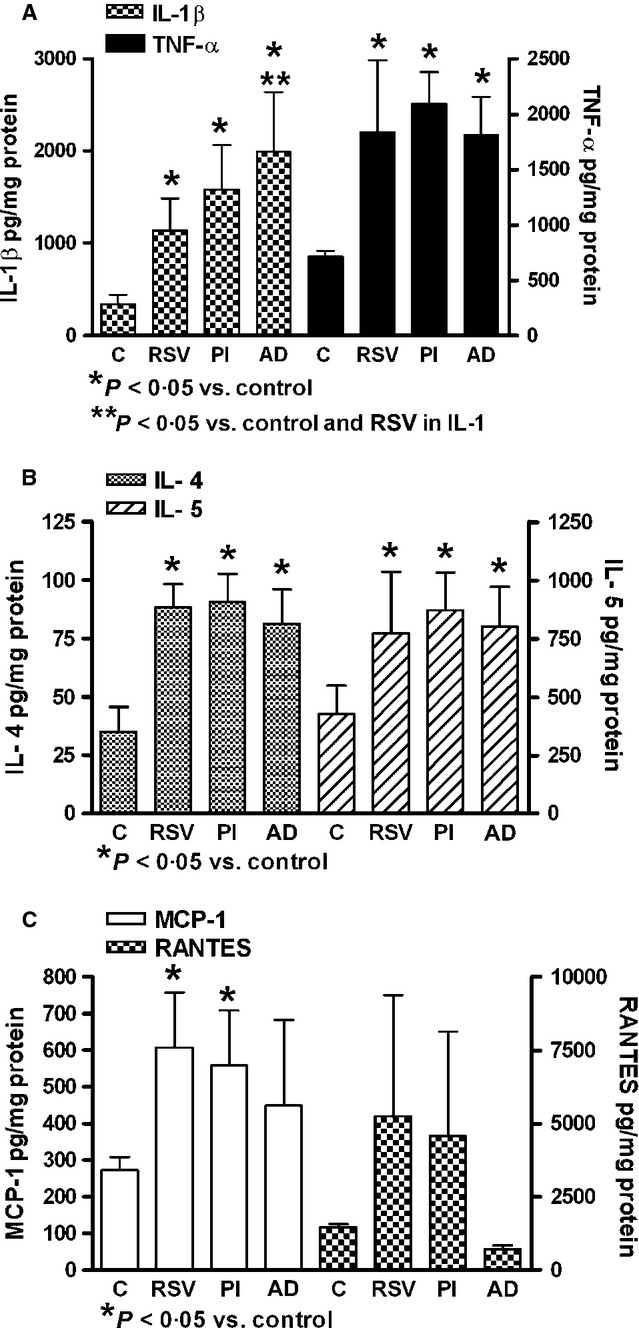
Interleukin-1 beta (IL-1β), tumor necrosis factor alpha (TNF-α), interleukin-4 (IL-4), interleukin-5, monocyte chemoattractant protein-1 (MCP-1), and regulated on activation normal T cell expressed and secreted (RANTES) expressions in human monocyte cultures infected by respiratory syncytial virus (RSV), parainfluenza virus (PI), or adenovirus (AD). C: Uninfected cultures. Healthy donors (*N* = 5).

*In vitro* infection of normal monocyte cultures by RSV, parainfluenza virus, or adenovirus induced increased cytokine concentrations in culture supernatants; only RSV and parainfluenza virus were capable of inducing significant increase of MCP-1 expression (Figure 4).

No significant increased expression of IL-8 was found in patient's serum (control: 29·55 ± 5·81; viral ARI: 73·58 ± 60·30 pg/mL) or in viral-infected monocyte cultures (control: 648·1 ± 164·4; RSV: 978·0 ± 239·4; parainfluenza: 1133·0 ± 250·5 and adenovirus: 961·3 ± 130·2 pg/mg protein). Expression of IL-4 was positively correlated with IL-5 expression (*r* = 0·4662; *P* < 0·001). There was no correlation between the studied serum cytokines and serum IgE levels. There was no association between the age of the donors and cytokine/chemokine production.

## Discussion

The respiratory tract is a frequent primary site of viral infections and may result in the development of acute respiratory distress syndrome (ARDS). The current understanding of the pathogenesis of ARDS suggests that the degree of inflammatory response and its sustained leukocyte activation may determine the clinical evolution of ARDS. During the lung inflammatory events, the alveolar compartmentalization could be lost, allowing the passage of cytokines released to the bloodstream by any other organ to the pulmonary endothelium or inversely from the lung to the bloodstream. These cytokines, especially IL-1, TNF-α, and IL-8, have important roles in the lung dysfunction.^7^ In this study, patients with viral ARI had increased serum levels of proinflammatory cytokines (IL-1β and TNF-α), Th2 cytokines (IL-4 and IL-5), and chemokines (MCP-1 and RANTES), suggesting complex immune interactions during viral respiratory infection. The source of those cytokines may be cells localized in lung tissue. In this regard, epithelial cells, mast cells, basophils, monocyte/macrophages, and lymphocytes could secrete diverse types of cytokines and chemokines (IL-1, TNF, IL-4, IL-5, IL8, MCP-1, RANTES) during lung tissue–virus interactions.^8^–^10^ However, in this study, monocyte cultures infected by RSV, adenovirus, or parainfluenza virus were capable of reproducing a similar cytokine and chemokine profiles to those observed in virus-infected patients, suggesting that in part, circulating monocytes could be the source of serum cytokines and chemokines. Accordingly, the chemokine and chemokine receptor inducer effect of RSV on monocyte/macrophage cultures has been reported.^11^ As alveolar compartmentalization could be lost during viral-induced lung injury,^7^ a combination of both lung and circulating monocyte cytokine and chemokine productions can contribute to the increased serum levels found in this study.

Patients with ARI were mainly infected by RSV. Human respiratory syncytial virus is a ubiquitous respiratory virus causing serious lower respiratory tract disease in infants and young children worldwide. Previous studies have shown that RSV infection modulates cytokine (IL-6, IL-1, TNF-α, IL-8, and GM-CSF), chemokine (IL-8, IP-10, MCP-1, and RANTES), and chemokine receptor expression *in vivo* and *in vitro,* suggesting that particular cytokine expression profiles may be an indicator of disease severity.^11^–^13^ Accordingly, in this study, serum from RSV-infected patients and RSV-infected monocyte cultures had increased levels of IL-1β, TNF-α, IL-4, IL-5, MCP-1, and RANTES. These effects probably are a common feature of respiratory virus infection, because similar serum cytokine/chemokine levels and viral cytokine inducer effect on monocyte cultures were observed in adenovirus and parainfluencia virus infections. In addition, infection with RSV could be a risk factor for further lung diseases, as the persistence of cytokines and chemokines in fully recovered RSV-infected patients can provide a substratum for the development of subsequent asthma.^14^

Regardless of cytokine production sources, they can have harmful effects on the lungs. The initial injury in lung tissue could release factors that contribute to inflammation, including chemokines (MCP-1), interleukins (IL-1β, IL-2, IL-4, IL-13), and prostaglandins (PGE_2_).^15^ This study found increased the expression of IL-1β, TNF- α, IL-4, IL-5, MCP-1, and RANTES in the serum of patients with viral respiratory infection. These cytokines could have a deleterious effect in the lung and other tissues. TNF-α can induce apoptosis in lung tissues with resulting in emphysema^16^ and activation of several proinflammatory events.^17^–^20^ IL-1 can induce neutrophilic inflammatory responses in respiratory disorders^21^ and release many other cytokines and chemokines.^22^,^23^ In addition, IL-1β induces eosinophil accumulation and it is a Th2 and B cell growth factor.^24^ In this regard, during this study the activation of Th2 profile was observed as shown by the increased production of IL-4 and IL-5. These cytokines increase allergic airway inflammation.^25^–^27^ Increased concentration of circulating chemokines found in asthmatic patients may be important in leukocyte recruitment to lung tissues. RANTES is involved in the chemoattraction of eosinophils, monocytes, and T-lymphocytes, and therefore, it has high relevance to asthma.^10^ MCP-1 is involved in the recruitment of regulatory and effector leukocytes (monocytes, lymphocytes, and basophils) into tissues.^9^ Therefore, chemokines could play an important role in asthma pathogenesis.

IgE has a multifunctional role in the pathogenesis of allergic inflammation. Beside its involvement in IgE-mediated degranulation of mast cells and basophils, it is also involved in the activation of macrophage/monocytes and the stimulation of Th2 cells. Both involve local infiltration of IL-4 and IL-5 secreting Th2-like cells, and both show pathologic evidence of epithelial damage, which likely serves to amplify tissue inflammation. In the case of asthma, bronchial epithelial damage (e.g., damage caused by viruses or eosinophil cationic proteins) and cytokine release from airway epithelium are believed to play an important role in the pathogenesis of airway inflammation.^28^ In this study, increased levels of IgE was found in patients with an asthmatic background, associated with increased production of circulating IL-4 and IL-5, suggesting a possible role of IgE in the increased levels of those cytokines and in the pathogenesis of asthma; however, correlation analysis failed to demonstrate statistical significance between IL-4 or IL-5 and IgE values.

Previous reports have shown differences in the bronchoalveolar lavage cytokine profiles between chronic obstructive pulmonary disease (COPD) and asthma. In this regard, the profile of cytokine expression in COPD is different from that in asthma, probably related to the type of cellular infiltrate observed in the two diseases.^3^ Thus, in asthma, an infiltration of eosinophils and Th2-cells involving IL-4, 5 and 13 production is usually found. In COPD, the neutrophil chemokine IL-8 and proinflammatory cytokines IL-1 and TNF-α play a more prominent role.^3^ In our study, the bronchoalveolar lavage cytokine profile was not analyzed; however, circulating cytokine profiles showed no differences in the expression of proinflammatory and Th2 cytokine profiles in both asthmatic and non-asthmatic patients, suggesting that the response during virus infection was not related to atopic status. In addition, levels of IgE were not correlated with Th2 (IL-4 and IL-5) expression. Only the serum levels of chemokines were observed to be increased in asthmatic patients, suggesting an increased cellular infiltration (monocytes, neutrophils, or eosinophils) at tissue level. Accordingly, the important role of chemokines in virus-associated asthma exacerbations has been reported.^13^ Previous reports have also shown different inflammatory response to RSV of asthmatic epithelium cultures compared with non-asthmatic epithelium.^12^ Comparative studies between asthmatic and non-asthmatic monocyte cultures were not performed in this study. However, cytokine/chemokine inducer effect of RSV and other viruses on monocyte cultures remained similar to the circulating cytokine profiles in asthmatic and non-asthmatic patients, suggesting a possible similar response of asthmatic and non-asthmatic monocyte cultures to virus infection. Nevertheless, we cannot rule out a different cellular response of viral-infected asthmatic monocyte cultures, because a different chemokine (MCP-1 and RANTES) profile was observed in RSV-treated monocyte cultures from asthmatic and non-asthmatic patients.^29^ As an unexpected finding in this study, we found similar levels of IL-4 in asthmatic and non-asthmatic patients, as asthmatic patients have a Th2 profile and RSV is a Th2-associated virus. We have not a clear explanation for this finding; however, previous exposure to RSV can sensitize non-asthmatic airways with further increased cytokine response. In this regard, RSV infection in neonates leads to inflammatory airway disease characterized by airway hyperreactivity, peribronchial, and perivascular inflammation, and subepithelial fibrosis in adults.^30^

In conclusion, the present study demonstrates the main characteristics of serum cytokine profiles in healthy, asthmatic, and non-asthmatic patients. While serum cytokine patterns were not different in non-asthmatic and asthmatic individuals, higher values of chemokines were observed in asthmatic patients. Furthermore, cytokine profiles observed in virus-monocyte cultures suggest a role of these cells in the increased circulating cytokines/chemokines in patients. The precise mechanism regarding how those systemic cytokines might be involved in driving viral respiratory infection processes remains elusive.
